# In-Hospital Peak Glycemia in Predicting No-Reflow Phenomenon in Diabetic Patients with STEMI Treated with Primary Percutaneous Coronary Intervention

**DOI:** 10.1155/2021/6683937

**Published:** 2021-01-08

**Authors:** Fang Liu, Rui Huang, Ya Li, Surui Zhao, Yue Gong, Zesheng Xu

**Affiliations:** Department 2 of Cardiology, Cangzhou Central Hospital, No. 16 Xinhua Road, Cangzhou, 061000 Hebei, China

## Abstract

Although percutaneous coronary intervention (PCI) significantly improves the prognosis for myocardial infarction, the no-reflow phenomenon is still the major adverse complication of PCI leading to increased mortality, especially for the patients with ST-segment elevation myocardial infarction (STEMI) combined with diabetes. To reduce the occurrence of no-reflow, prognostic factors must be identified for no-reflow phenomenon before PCI. A total of 262 participants with acute STEMI and diabetes were recruited into our cardiovascular center and underwent primary PCI for the analyses of prognostic factors of no-reflow. The patients were divided into two groups according to thrombolysis in myocardial infarction (TIMI): the normal flow and no-reflow groups, and related factors were analyzed with different statistical methods. In the present investigation, the in-hospital peak glycemia was significantly higher in the no-reflow group than the normal flow group, while more narrowed vessels, higher level of initial TIMI flow, were observed in the patients of the no-reflow group. A multivariate logistic regression analysis further demonstrated that peak glycemia was an independent predictor for no-reflow in the diabetic patients with STEMI. Our data indicated the importance of the proper control of glucose before PCI for the diabetic patients with STEMI before PCI to reduce the occurrence of the no-reflow after operation.

## 1. Introduction

Even though the therapeutic methods for patients with acute coronary syndrome (ACS) and stable coronary artery disease (CAD) have been substantially developed, angiographic optimal percutaneous coronary intervention (PCI) is still the preferred treatment for these patients, especially for patients with ST-elevation myocardial infarction (STEMI), a common form of acute myocardial infarction (AMI) [1–3]. However, no-reflow, the major adverse complication of PCI, causes increased mortality due to poor healing of the infarct, adverse left ventricular remodeling and congestive heart failure [4–6]. Tissue perfusion should occur for patients after PCI; however, about 1-3% of the patients encounter a no-reflow in the first 2 hours after PCI [6–11]. Although the occurrence of no-reflow is rare among patients with PCI, the serious complication of reperfusion in patients with STEMI leads to poor prognosis and increased mortality [12–14]. Therefore, to illustrate the prognostic factors of no-reflow phenomenon before PCI in patients with AMI is critically useful to provide guidance for surgeons and to prevent the occurrence of no-reflow.

Recently, an increasing number of cardiovascular risk factors are reported as potential predictive events to reduce the incidence of NR before the procedure, including hypertension control [15, 16] and use of statins [17] before the PCI. Studies demonstrated that optimal blood sugar control before PCI could significantly reduce the increased rate of no-reflow for patients with diabetes [15, 18]. However, the precise management of hyperglycemia in patients with diabetes is still lacking, and the range of in-hospital peak glycemia for the prognostic role of no-reflow has not yet been defined in patients with STEMI combined with diabetes.

The aim of this investigation was to reveal the association between hyperglycemia defined by in-hospital peak glycemia and the no-reflow phenomenon in STEMI patients with diabetes and to assess the prognostic role of peak glycemia in clinic to reduce no-reflow incidence and improve prognosis.

## 2. Methods

The AMI was diagnosed using the criteria of the “Guidelines for the Diagnosis and Treatment of Acute Myocardial Infarction” formulated by the annual Chinese Medical Association Cardiology Branch, the editorial board of the Chinese Journal of Cardiology, the editorial board of the Chinese Circulation Journal, and the international guidelines formulated by the American College of Cardiology [19]. The STEMI patients were first evaluated by the criteria of the classic symptoms of coronary ischemia and then diagnosed with a 1 mm ST-segment elevation in the inferior lead, or a 2 mm ST-segment elevation of the anterior chest lead, or on the presence of a new left bundle branch block. The diagnosis of diabetes is based on the criteria of the 2017 American Diabetes Association [20]. The TIMI (thrombolysis in myocardial infarction) was used to semiquantitatively assess coronary artery perfusion. The reflow was defined as complete perfusion of coronary after primary PCI with less than 20% residual stenosis in epicardial coronary arteries, whereas blood flow less than TIMI flow II in the absence of dissection, vasospasm, and stenosis in the final angiogram was identified as no-reflow [21, 22].

Blood samples were obtained before PCI, and the following parameters were measured: heart rate, blood pressure, hemoglobin, white blood cell count, CPK-MB (creatine phosphokinase, myocardial band), cTnI (cardiac troponin I), C-reactive protein, GFR (glomerular filtration rate, determined by the measurement of creatinine), TC (total cholesterol), TG (triglyceride), LDL-C (low-density lipoprotein cholesterol), and HDL-C (high-density lipoprotein cholesterol). A transthoracic two-dimensional echocardiography was used to assess the left ventricular ejection fraction (LVEF). Glycemia was assessed three times a day for the patients during hospital stay, and the peak values were considered for the study.

### 2.1. Study Population

From January 2017 to January 2019, a total of 262 consecutive patients with acute STEMI and history of diabetes were admitted to our cardiovascular center and underwent primary PCI. All the participant was stratified into two groups: the normal reflow (120 patients) and the no-reflow (142 patients). The informed consent was obtained from all patients, and this study was approved by the local ethics committee of Cangzhou Central Hospital. The TIMI grade was determined by two senior experts of cardiac interventional according to coronary angiography, who were blind to the group assignment and the design of the experiments.

Patients with the following criteria were recruited into this study: (1) age ≥ 18 years old, (2) patients with chest pain and chest tightness in the emergency department, (3) STEMI diagnosis based on “Acute Myocardial Infarction Diagnosis and Treatment Guidelines”, (4) within 24 hours from symptom onset, (5) written informed consent of PCI, and (6) patients diagnosed with the combination of diabetes. Patients with the following criteria were excluded from this study: (1) refused PCI; (2) diagnosed with STEMI in other hospitals; (3) STEMI occurred during the hospital stay; (4) the reperfusion occurred by thrombolysis before the hospitalization; (5) a history of an unprotected left main artery with severe liver and kidney diseases or coronary artery bypass grafting; (6) preexcitation syndrome; (7) severe dissection, thromboembolism in other parts, or vasospasm; and (8) known malignancy.

### 2.2. Statistical Analysis

The SPSS version 13.0 was used for the statistical analysis of this study. Student's *t* test was used to compare the duration of diabetes, preoperative blood glucose level, coronary angiography blood flow, and coronary artery CT stenosis level between two groups. Categorical data are represented as frequencies and percentages and compared with a *χ*^2^ test. Continuous variables without normal distribution were reported as median (25–75th percentile) with the Mann-Whitney *U* test. The analysis for the data of no-reflow and the death phenomenon were performed by the *χ*^2^ test. *p* < 0.05 was considered statistically significant. Variables associated with no-reflow were calculated with the odds ratio and 95% confidence interval. Conditional logistic regression analyses were used to determine the independent predictors of the no-reflow phenomenon. The receiver operating characteristic (ROC) curve was used to assess the accuracy and best cut-off values of in-hospital peak glycemia for the prognostic impact of no-reflow phenomenon in patients with STEMI combined with diabetes after PCI.

## 3. Results

A total of 262 patients with STEMI combined with diabetes were recruited for the study, which were divided into two groups according to the TIMI flow grade after primary PCI. The no-reflow group comprised of 142 participants with flow grade 0-1, and the normal flow comprised of 120 participants with TIMI flow grade 2-3. The patients were older in the no-reflow group than the normal reflow group (*p* = 0.004, Table 1) according to the demographic data. The prevalence of hypertension was significantly higher in the no-reflow group (Table 1), with higher admission glucose in this group as well (Table 1). There were more patients in the no-flow group with Killip class > 1 on admission than the normal flow group (26.8% vs. 17.5%, *p* = 0.013, Table 1). Higher levels of peak CK-MB (*p* < 0.001) and C-reactive protein (*p* = 0.005) were observed in patients with no-reflow than those with normal reflow. In particular, the level of in-hospital peak glycemia was significantly higher in patients with no-reflow than patients with normal reflow (2.35 ± 0.86 vs. 1.76 ± 0.67, *p* < 0.001, Table 1). Besides, other clinical, laboratory, and procedural parameters showed statistical differences between these two groups, including white blood cell count, estimated GFR (glomerular filtration rate), TG, and LDL-C (Table 1).

The angiographic characteristics showed statistical differences between the two groups in terms of the number of narrowed vessel and level of initial TIMI flow (Table 2). Specifically, there were significantly more participants in the no-reflow group with multivessel disease than the normal flow group (Table 2). The TIMI flow level was 0 for most patients (79.6%) with no-reflow, and less patients were observed with a TIMI flow > 0 than the normal reflow group (*p* = 0.002, Table 2). In addition, the type of infarct-related artery, the strategy of PCI procedure, and the length of stent were similar in the two groups (Table 2). In summary, all these analyses suggested that there were more patients in the no-reflow group with numerous narrowed vessel and high level of initial TIMI flow, which were the main factors of a successful PCI procedure. As depicted in previous data, the in-hospital peak glycemia showed a significant difference in the no-reflow and normal reflow groups (Table 1). To investigate whether the values of in-hospital peak glycemia were associated with the occurrence of no-reflow, the patients were further divided into three groups for the re-reflow and normal reflow groups according to the in-hospital peak glycemia: ≤1.40 g/L, 1.41-1.80 g/L, and >1.8 g/L. As shown in Table 3, there were more patients with peak glycemia > 1.8 g/L in the no-reflow group than the normal reflow group (86.6% vs. 57.5%, *p* < 0.001, Table 3). Moreover, the data also demonstrated that higher peak glycemia levels were associated with higher levels of admission glucose (*p* < 0.001, Table 3) and peak glucose (*p* < 0.001, Table 3). Taken together, all these data implied that the values of peak glycemia were highly associated with the no-reflow phenomenon in diabetic STEMI patients after PCI.

**Figure 1:**
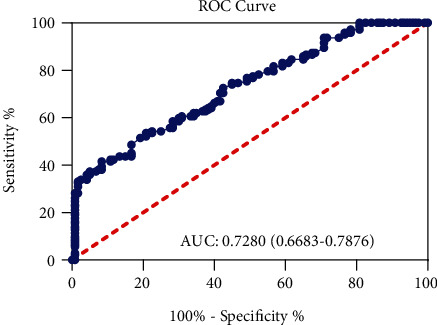
ROC curve analysis for in-hospital peak glycemia for prediction of no-reflow phenomenon after pPCI in patients with STEMI combining with diabetes.

Univariate and multivariate logistic regression analyses were performed to assess the effects of the various variables with an unadjusted *p* < 0.05 in the no-reflow group. In addition to in-hospital peak glycemia (*p* < 0.001, Table 4), the result of the univariate analysis showed that age, hypertension, estimated GFR, peak CK-MB, and C-reactive protein were closely related to the no-reflow phenomenon. Moreover, a ROC statistical analysis demonstrated that the value of in-hospital peak glycemia > 2.215 g/L had 58.45% sensitivity and 71.67% specificity for predicting the no-reflow status in patients with STEMI combined with diabetes (*p* < 0.001, Figure 1).

To reveal the independent contributions of these variables, a further multivariate logistic regression analysis was performed for the clinical factors showing significance in the univariate analysis. The data showed that hypertension, estimated GFR, in-hospital peak glycemia, peak CK-MB, and C-reactive protein remained as independent predictors of no-reflow after primary PCI in patients with STEMI and diabetes (Table 4).

## 4. Discussion

In the present study, we observed higher levels of in-hospital peak glycemia in the no-reflow group compared with the normal reflow groups (*p* < 0.001, Table 1) and more narrowed vessels and higher level of initial TIMI flow were demonstrated in the patients of the no-reflow group. To our knowledge, this study is the first to demonstrate that in-hospital peak glycemia is an independent predictor for no-reflow in diabetic STEMI patients, and the values of peak glycemia showed close correlation with the no-reflow phenomenon in diabetic STEMI patients after PCI. Moreover, hypertension, estimated GFR, peak CK-MB, and C-reactive protein were the other predictors of no-reflow.

The no-reflow phenomenon is mainly caused by the distal embolization and reperfusion injury, and the patients might complain of chest pain with elevated ST-segment in the electrocardiogram [11, 23]. The perfusion of the coronary blood is the most important clinical indicator for the assessment of successful PCI in patients with STEMI. The good recovery of the microvascular function and the reperfusion determines the functional and clinical outcomes after AMI [7, 18, 24]. Therefore, emerging studies are dedicated to reveal the predictors of no-reflow in the STEMI patients before PCI [15–17]. Investigations have found that hyperglycemia and peak glycemia are associated with the highest mortality in patients with or without diabetes [16, 25]. Hyperglycemia was highly associated with AMI and the increased mortality in patients with diabetes mellitus after AMI [15, 26–28]. However, to our knowledge, there is no study conducted to reveal the relationship of the peak glycemia for the precise management of hyperglycemia, specifically in diabetic patients with STEMI [29]. In the present investigation, we found that not all of the diabetic patients face a higher risk of no-reflow, and proper control of the peak glycemia would be helpful for the prevention of no-flow.

Our data found that patients with a high level of peak glycemia (>1.8 g/L) were more likely to have no-reflow (86.6%, Table 3), whereas patients with a lower level of peak glycemia (<1.8 g/L) exhibited relatively lower occurrence rates of no-reflow (9.2% and 4.2% for the groups 1.41-1.80 and ≤1.40 g/L, respectively). A further logistic regression analysis showed that the in-hospital peak glycemia might be an independent predictor of no-reflow. In summary, our data indicated that proper in-hospital glycemic control indicated by the values of in-hospital peak glycemia (g/L) might be a clinical guideline to reduce the probability of no-reflow for diabetic patients with STEMI. We further observed that the level of in-hospital glycemia showed high correlation with admission glucose and peak glucose (Table 3).

Even though the American College of Cardiology and European Society of Cardiology appeal to eliminate the routine detection of CK-MB tests, which are suggested to be replaced by the test of cardiac troponin for ACS patients, the majority of the hospitals are still choosing the CK-MB test as the cardiac biomarker clinically [30]. Interestingly, our study showed that most patients in the no-reflow group exhibited a dramatically higher level of peak CK-MB than the normal reflow group (median 135.2 vs. 91.5, Table 1), which suggested the importance and necessity for the detection of CK-MB in diabetic patients with STEMI. In addition, a significantly higher inflammatory activation (as indicated by higher levels of white blood cell count and C-reactive protein, Table 1) was also documented in the study, which reminded us to pay attention to these factors for their potential prognostic role of no-reflow for diabetic patients after primary PCI.

The exact mechanism of how hyperglycemia affects the occurrence of no-reflow during primary PCI is unclear, and several potential pathologic mechanisms have been reported to explain the relationship between hyperglycemia and the no-reflow phenomenon. A report demonstrated that levels of hs-CRP and IL-6 before and after PCI showed high association with adverse cardiac events and major adverse cardiac events [31–35]. Interestingly, we also observed a higher inflammatory activation indicated by higher levels of white blood cell count and C-reactive protein. Therefore, the inflammatory factors should be paid more attention to before and after PCI.

Besides hs-CPR and IL-6, studies revealed that cardiac diseases are highly related to other inflammatory factors [36–38], such as increased oxidative stress [39], procoagulant activity [40], and adhesion molecules [41]. The homeostasis between inflammation/oxidative stress and cytokine milieu might be the core factors for the prevention of restenosis after PCI. However, our study did not involve any mechanism of hyperglycemia for no-reflow and further experiments are needed for the investigation of a mechanism related to hyperglycemia and no-reflow in the future. Some other limitations also existed in this investigation. The most important one is that the cohort of participants in the present study is relatively small due to the research conditions in the hospital, complicating the credibility and generalization of the result in the present study. Therefore, more diabetic patients with STEMI diagnosed in this hospital should be recruited in the future to verify our results.

## 5. Conclusion

In summary, for patients with STEMI combined with diabetes, in-hospital peak glycemia is an independent predictor for no-reflow after primary PCI. Our data underscore the clinical importance of in-hospital glycemia and proper control of glucose before PCI in reducing the occurrence of no-reflow for patients with STEMI and diabetes. Moreover, the estimated GFR is demonstrated as another independent prognostic factor of no-reflow in this study and further study should be performed to further study the relationship between GFR and no-reflow.

## Figures and Tables

**Table 1:** The baseline characteristics and laboratory results of patients classified according to no-reflow.

	Normal reflow (*n* = 120)	No-reflow (*n* = 142)	*p* value
Age (years)	62.3 ± 8.9	68.1 ± 11.2	0.004
Female gender	19 (15.8%)	24 (16.9%)	0.816
Hypertension	51 (42.5%)	84 (59.2%)	<0.001
Smoking	32 (26.7%)	43 (30.3%)	0.519
Family history of CAD	28 (23.3%)	39 (27.5%)	0.445
Heart rate (bpm)	78.9 ± 11.7	80.6 ± 10.3	0.085
Systolic blood pressure (mmHg)	131.78 ± 20.32	133.47 ± 21.63	0.294
Diastolic blood pressure (mmHg)	74.42 ± 14.05	75.98 ± 13.25	0.366
Admission LVEF (%)	50.6 ± 4.8	51.7 ± 5.2	0.135
In-hospital peak glycemia (g/L)	1.76 ± 0.67	2.35 ± 0.86	<0.001
Admission glucose (g/L)	1.58 ± 0.69	2.05 ± 0.78	0.024
Killip class > 1 on admission	21 (17.5%)	38 (26.8%)	0.013
White blood cell count (10^3^/*μ*L)	11.92 ± 3.65	13.34 ± 3.81	0.004
Estimated GFR (mL/min/1.73 m^2^)	82.6 ± 19.5	69.4 ± 17.4	0.026
Hemoglobin (g/dL)	13.3 ± 1.5	12.6 ± 2.1	0.218
TC (mg/dL)	162.8 ± 38.2	159.4 ± 40.4	0.541
TG (mg/dL)	96.4 ± 34.6	108.5 ± 30.5	0.008
LDL-C (mg/dL)	102.6 ± 24.7	99.8 ± 27.1	0.094
HDL-C (mg/dL)	38.2 ± 10.8	36.1 ± 11.2	0.227
Peak cTnI (U/L)	8.32 ± 3.21	8.69 ± 3.98	0.308
Peak CK-MB (U/L)	91.5 (65.3–108.7)	135.2 (73.5–196.6)	<0.001
C-reactive protein (mg/dL)	9.4 (5.3–18.1)	14.3 (7.6–27.2)	0.005

CAD: coronary artery disease; LEVF: left ventricular ejection fraction; GFR: glomerular filtration rate; TC: total cholesterol; TG: triglyceride; LDL-C: low-density lipoprotein cholesterol; HDL-C: high-density lipoprotein cholesterol; cTnI: cardiac troponin I; CK-MB: creatine kinase myocardial band. The data presented are mean ± SD or *n* (percentage) or median (95% confidence interval (CI)). The comparison of data between the two groups was done by the Student *t* test or Fisher's exact test.

**Table 2:** Angiographic findings of patients classified according to no-reflow.

	Normal reflow (*n* = 120)	No-reflow (*n* = 142)	*p* value
Number of narrowed vessels			
Single vessel	63 (52.5%)	50 (35.2%)	0.005
Multivessel	57 (47.5%)	92 (64.8%)	
Initial TIMI flow			
0	70 (58.3%)	113 (79.6%)	0.002
1	31 (25.8%)	18 (12.7%)	
2	13 (10.9%)	5 (3.5%)	
3	6 (5%)	6 (4.2%)	
Infarct-related artery			
LAD	45 (37.5%)	69 (48.6%)	0.160
RCA	47 (39.2%)	42 (29.6%)	
CFX	28 (23.3%)	31 (21.8%)	
PCI procedure			
Balloon angioplasty	13 (10.8%)	18 (12.7%)	0.639
Balloon+stenting	91 (75.8%)	110 (77.4%)	
Primary stenting	16 (13.4%)	14 (9.9%)	
Stent length (mm)	17.51 ± 3.67	18.13 ± 3.89	0.315

TIMI: thrombolysis in myocardial infarction; LAD: left anterior descending artery; CFX: circumflex coronary artery; RCA: right coronary artery; PCI: percutaneous coronary intervention. The data presented are mean ± SD or *n* (percentage) or median (95% confidence interval (CI)). The comparison of data between the two groups was done by the Student *t* test or Fisher's exact test.

**Table 3:** In-hospital peak glycemia and the occurrence of no-reflow.

	In-hospital peak glycemia (g/L)			*p* value
	≤1.40	1.41-1.80	>1.8	
Normal reflow	15 (12.5%)	36 (30.0%)	69 (57.5%)	<0.001
No-reflow	6 (4.2%)	13 (9.2%)	123 (86.6%)	
Admission glucose (g/L)	1.17 (1.07–1.28)	1.52 (1.43–1.64)	2.12 (1.84–3.04)	<0.001
Peak glucose (g/L)	1.29 (1.11–1.39)	1.67 (1.50–1.78)	2.41 (1.93–2.82)	<0.001

The data presented are *n* (percentage) or median (95% confidence interval (CI)). The comparison of data among the three groups was done by one-way ANOVA or Fisher's exact test.

**Table 4:** Effects of various variables on no-reflow in univariate and multivariate logistic regression analyses.

	Univariate analysis		Multivariate analysis	
	Odds ratio, 95% CI	*p* value	Odds ratio, 95% CI	*p* value
Age (years)	1.029 (1.010-1.059)	0.003	1.056 (0.995–1.049)	0.246
Gender (male)	1.083 (0.442-1.982)	0.684		
Smoking	0.682 (0.386-1.035)	0.534		
Family history of CAD	0.624 (0.421–0.916)	0.316		
Hypertension	1.123 (0.895–1.389)	0.038	1.098 (0.796-1.337)	0.043
Estimated GFR	0.975 (0.937–0.994)	0.004	0.986 (0.971–1.032)	0.012
In-hospital peak glycemia (g/L)	1.536 (1.345–1.685)	<0.001	1.448 (1.341–1.675)	<0.001
TG (mg/dL)	1.084 (0.768-1.362)	0.381		
Killip class > 1 on admission	1.405 (0.813-1.767)	0.156		
White blood cell count, (10^3^/*μ*L)	1.224 (0.818-1.405)	0.339		
Peak CK-MB (U/L)	1.389 (1.124-1.693)	<0.001	1.265 (1.093-1.472)	<0.001
C-reactive protein (mg/dL)	1.146 (1.045-1.209)	0.006	1.095 (1.017-1.184)	0.017

CAD: coronary artery disease; GFR: glomerular filtration rate; TG: triglyceride; CK-MB: creatine kinase myocardial band.

## Data Availability

The data used to support the findings of this study are available from the corresponding author upon request.
